# A Potential Protein Adjuvant Derived from *Mycobacterium tuberculosis* Rv0652 Enhances Dendritic Cells-Based Tumor Immunotherapy

**DOI:** 10.1371/journal.pone.0104351

**Published:** 2014-08-07

**Authors:** Seung Jun Lee, Sung Jae Shin, Moon Hee Lee, Min-Goo Lee, Tae Heung Kang, Won Sun Park, Byoung Yul Soh, Jung Hee Park, Yong Kyoo Shin, Han Wool Kim, Cheol-Heui Yun, In Duk Jung, Yeong-Min Park

**Affiliations:** 1 Department of Immunology, Lab of Dendritic Cell Differentiation & Regulation, School of Medicine, Konkuk University, Chungju, South Korea; 2 Department of Microbiology, Institute for Immunology and Immunological Diseases, Brain Korea 21 PLUS Project for Medical Science, Yonsei University College of Medicine, Seoul, South Korea; 3 Department of Physiology, College of Medicine, Korea University, Seoul, South Korea; 4 Department of Physiology, School of Medicine, Kangwon National University, Chuncheon, South Korea; 5 Department of Biochemistry, College of Medicine, Seonam University, Namwon, Jeonbuk, South Korea; 6 Division of Biotechnology, Advanced Institute of Environment and Bioscience, College of Environmental & Bioresources Sciences, Chonbuk National University, Iksan, South Korea; 7 Department of Pharmacology, College of Medicine, Chung-Ang University, Seoul, South Korea; 8 Department of Agricultural Biotechnology, and Research Institute for Agriculture and Life Sciences, Seoul National University, Seoul, South Korea; Shanghai Jiao Tong University School of Medicine, China

## Abstract

A key factor in dendritic cell (DC)-based tumor immunotherapy is the identification of an immunoadjuvant capable of inducing DC maturation to enhance cellular immunity. The efficacy of a 50S ribosomal protein L7/L12 (rplL) from *Mycobacterium tuberculosis* Rv0652, as an immunoadjuvant for DC-based tumor immunotherapy, and its capacity for inducing DC maturation was investigated. In this study, we showed that Rv0652 is recognized by Toll-like receptor 4 (TLR4) to induce DC maturation, and pro-inflammatory cytokine production (TNF-alpha, IL-1beta, and IL-6) that is partially modulated by both MyD88 and TRIF signaling pathways. Rv0652-activated DCs could activate naïve T cells, effectively polarize CD4^+^ and CD8^+^ T cells to secrete IFN-gamma, and induce T cell-mediated-cytotoxicity. Immunization of mice with Rv0652-stimulated ovalbumin (OVA)-pulsed DCs resulted in induction of a potent OVA-specific CD8^+^ T cell response, slowed tumor growth, and promoted long-term survival in a murine OVA-expressing E.G7 thymoma model. These findings suggest that Rv0652 enhances the polarization of T effector cells toward a Th1 phenotype through DC maturation, and that Rv0652 may be an effective adjuvant for enhancing the therapeutic response to DC-based tumor immunotherapy.

## Introduction

Dendritic cells (DCs) are the most important regulators of naïve cells during the generation of T cell-mediated immune responses, and DCs are the most potent antigen-presenting cells (APCs) [1]. Because of their central role in modulating cellular immunity, DCs are promising as potent adjuvants for the induction of tumor-specific helper and cytotoxic T cells in tumor-bearing hosts [2], [3]. Immunization with DCs loaded *ex* *vivo* with tumor-associated antigens (Ags) is a promising approach for inducing effective antitumor immunity [4]. This approach has been successfully applied to vaccinations to activate cytotoxic T lymphocyte (CTL) responses [5]–[8]. Early clinical trials have shown that tumor vaccine therapies using DCs activated tumor Ags alone do not generate an effective antitumor immune response, even though this strategy has been successful in the treatment of tumors in murine models [5], [6], [9]–[11]. Several mechanisms may account for the limited effectiveness of DC vaccine induced immunity against tumors. One possibility is that insufficient numbers of CTLs, the effector cells that are considered optimal for causing tumor regression, are activated in response to vaccination with tumor Ag loaded DCs [12]. To overcome this limitation of DC-based antitumor immunotherapeutic strategies, several factors related to *in* *vitro* DC manipulation are important for inducing a powerful immune response. Because DC maturation is critical for the generation of effective immunity in therapeutic studies [13], it is important to consider the regulation of DC maturation. Immunostimulatory adjuvants used to enhance DC maturation are required to generate endogenous CTL responses and tumor elimination.

The use of Toll-like receptor (TLR)-mediated DC activation may be an effective cancer treatment strategy because TLR activation usually induces Th1 responses [14], [15]. When a ligand is bound by a TLR, signaling occurs via two adaptor molecules in the cytoplasm: myeloid differentiation primary response protein 88 (MyD88) and Toll/IL-1R domain-containing adaptor inducing IFNbeta (TRIF). TRIF is used for signaling by all TLRs except for TLR3 [16]. Synthetic agonists of TLR3, TLR4, TLR5, TLR7, TLR8, and TLR9 have been identified as suitable immunostimulants [17]–[22], and simultaneous engagement of more than one TLR have resulted in better immune stimulation *in* *vitro* [23]. Based on the efficacy of TLR agonists used thus far, the use of TLR ligands as adjuvants in humans is likely to increase in the future. Thus, DC activation by various microbes via TLR signaling is the critical link between innate and adaptive immunity and is crucial for generating protective immune responses.

Proteins that polarize the activated immune systems toward a Th1 response are expressed by specific bacteria of the genus *Mycobacterium* and related genera, and these adjuvants are predicted to be crucial components of immunotherapeutic strategies [24]. A significant amount of effort has been focused on characterizing all mycobacterial proteins to identify antigenic proteins that can be used as vaccine candidates for tuberculosis therapy [24]. *Mycobacterium tuberculosis* ligands such as PE-PGRS and LprA are known to interact with and activate APCs, specifically DCs [25], [26]. However, the mechanisms mediating the interaction between these mycobacterial proteins and the host, which contribute to the development of vaccine adjuvants for diseases, have not been adequately clarified. It has been suggested recently that *M. tuberculosis* heparin-binding hemagglutinin (HBHA) is a potent immune adjuvant with potential applications in antitumor immunotherapy [27]. Together, these reports demonstrate the potential usefulness of mycobacterial proteins in DC-based antitumor immunotherapy.

*M. tuberculosis* protein Rv0652, the 50S ribosomal protein (rp) L7/L12 (rplL) [28], is a mixture of the L7 and L12 components of heat-stable purified protein derivative proteins which are trigger a strong delayed-type hypersensitivity reaction [29]. The rplL protein is involved in translation factor binding, GTP-hydrolysis, and translocation [30], [31]; however, little is known regarding the function of Rv0652 or the mechanism by which this protein influences innate and adaptive immunity.

The biological activity and cellular immunity of *M. tuberculosis* Rv0652 in DCs and their potential as adjuvants in DC-based antitumor immunotherapy are described. Rv0652 is shown to be a potent TLR4 agonist, inducing the MyD88 and TRIF signaling pathways, and possibly enhancing DC activation and Th1 polarization. Notably, Rv0652 mediated a strong induction of Ag-specific, CD8^+^ class I-restricted, CTL responses, and E.G7 tumor regression *in* *vivo*. These results highlight the potential use of Rv0652 for DC-based antitumor immunotherapy.

## Materials and Methods

### Mice

Male 6 to 8-week-old C57BL/6 (H-2K^b^ and I-A^b^) mice were purchased from the Korean Institute of Chemistry Technology (Daejeon, Korea). C57BL/6 OT-I and C57BL/6 OT-II T-cell receptor transgenic mice, C57BL/6J TLR2 knockout mice (*TLR2* ^−*/*−^; B6.129-Tlr2^tm1Kir^/J), C57BL/10 TLR4 knockout mice (*TLR4* ^−*/*−^; C57BL/10ScNJ), MyD88 knockout mice, and TRIF-deficient mice aged 6–8 weeks were purchased from Jackson Laboratory (Bar Harbor, ME). Mice were housed in a specific pathogen-free environment within an animal facility for at least 1 week before the experiment. All animal procedures were performed in accordance with the protocols approved by the Konkuk University Institutional Animal Care and Committee and the recommendations for the proper use and care of laboratory animals (IACUC number: KU13047).

### Cell lines

EL4, a thymoma-derived cell line from the C57/BL/6 (H-2K^b^ and I-A^b^) mouse, and E.G7, an ovalbumin (OVA)-expressing EL4 variant, were purchased from the American Type Culture Collection (Manassas, VA) and cultured in RPMI 1640 medium supplemented with 10% heat-inactivated FBS, 100 U/ml penicillin, 100 ug/ml streptomycin, and 10 mM l-glutamine (all purchased from Invitrogen, Carlsbad, CA) at 37°C in 5% CO_2_.

### Reagents and antibodies

Recombinant mouse granulocyte-macrophage colony-stimulating factor (GM-CSF), interleukin (IL)-4, CCL19, and the FITC-annexin V/propidium iodide kit were purchased from R&D Systems (Minneapolis, MN). Dextran-FITC (40, 000 Da) was purchased from Sigma-Aldrich (St. Louis, MO). Lipopolysaccharide (LPS, from *Escherichia coli* O111: B4) was purchased from Invivogen (San Diego, CA). H-2K^b^-restricted OVA peptide (OVA_257–264_) and H-2b-restricted OVA peptide (OVA_323–339_) were synthesized by Peptron (Daejeon, Korea). The following FITC- or PE-conjugated monoclonal Abs were purchased from BD Biosciences (San Jose, CA): CD11c (HL3), CD80 (16-10A1), CD86 (GL1), Iab beta-chain (AF-120.1), H-2K^b^ (AF6-88.5), CCR7 (CD197), IL-10 (JESS-16E3), and IL-12p40/p70 (C15.6). Alexa568 conjugated anti-mouse IgG antibodies were purchased from Invitrogen (Eugene, OR). FITC-conjugated Mouse IgG Abs and cytokine ELISA kits for murine IL-1beta, IL-2, IL-6, IL-10, IL-12p70, TNF-alpha, and IFN-gamma were purchased from eBiosciences (San Diego, CA).

### Expression and purification of recombinant Rv0652 protein

Recombinant Rv0652 (rRv0652) protein was produced using pET28a vector (Promega, Madison, WI) and an *Escherichia coli* expression system, as recently described (26). Briefly, *rplL* encoding Rv0652 was amplified by polymerase chain reaction (PCR) using *M. tuberculosis* H37Rv genomic DNA (ATCC27294) as template and the following primers: forward, 5′-GGG CCC GGA TCC ATG GCA AAG CTC TCC-3′; and reverse 5′-GGG CCC GAA TTC CTT GAC GGT GAC GGT-3′. The PCR product was cut with *Bam*HI and *Eco*RI and then inserted into pET28a vector cut with the same restriction enzymes. The recombinant plasmid was transformed into *E*. *coli* BL21 cells carrying bacteriophage DE3 for protein overexpression.

### DC generation and culture

DCs were isolated and cultured as recently described [32]. In some experiments, DCs were labeled with bead-conjugated anti-CD11c mAb and positively selected (>95%) according to the manufacturer’s instructions (LS columns; Miltenyi Biotec, Auburn, CA).

### Therapeutic implanted tumor experiments

Mice were injected subcutaneously (s.c.) into the right lower back with EL4 or E.G7 thymoma cells (2×10^6^ cells), followed by tail vein injection of immature and Rv0652-treated DCs (1×10^6^ cells), some of which were pulsed with OVA (1 ug/ml) on days 1, 3, and 5 after tumor inoculation. Groups of tumor-bearing mice were treated with PBS, iDCs (immature DCs), DCs-OVA (DCs pulsed with OVA peptide), LPS-DCs-OVA (LPS-treated DCs pulsed with OVA peptide), or Rv0652-DCs-OVA (Rv0652-treated DCs pulsed with OVA peptide). Tumor size was measured every 2 days, and tumor mass was calculated as: V = (2*A*×*B*)/2, where *A* is the length of the short axis and *B* is the length of the long axis.

### Confocal laser scanning microscopy

Cells were fixed with 4% paraformaldehyde (PFA), then stained with anti-mouse Rv0652 and Alexa568 conjugated anti-mouse IgG antibodies overnight at 4°C to detect Rv0652. Fluorescence intensity was analyzed using a confocal laser scanning microscope (Zeiss, Germany). Images were acquired using LSM510 Meta software and processed using the LSM image examiner.

### Antigen uptake and migration of DCs

Antigen uptake and *in* *vitro* and *in* *vivo* chemotaxis were performed as previously described (26).

### Mixed lymphocyte reaction

In brief, DCs were incubated with Rv0652 or LPS in the presence or absence of OVA_257–264_ (0.5 ug/ml) or OVA_323–339_ (0.5 ug/ml) for 24 h. CFSE-labeled OT-I and OT-II T-cells were seeded in triplicate wells at a density of 1×10^5^ cells per well, together with DCs at a density of 1×10^4^ cells per well, in U-bottomed 96-well microtiter culture plates (Nunc). Cells were harvested after 96 h, stained with Cy5-labeled anti-CD8 and anti-CD4 monoclonal Abs, and analyzed by flow cytometry (BD Biosciences).

### *Ex vivo* CTL assay and ELISA for IFN-gamma and IL-2

BALB/c mice were injected with PBS, untreated DCs (iDC), DCs pulsed with OVA_257–264_ (DC-OVA), or Rv0652-treated DCs pulsed with OVA_257–264_ (Rv0652-DC-OVA) on days 1 and 7. Spleen cells (2×10^6^ per well) from injected mice (day 10) were restimulated with 10 ug/ml OVA_257–264_ for 7 days in 6-well plates, then co-cultured with EL4 [1×10^6^, 0.5 uM CFSE-stained (CFSE^low^)] and E.G7 [1×10^6^, 10 uM CFSE-stained (CFSE^high^)] cells. After 2 days, mixed cells were analyzed for cytotoxicity against tumor cells by flow cytometry. After 1 day, the culture supernatant was used for IFN-gamma and IL-2 ELISAs.

### Statistical analysis

Statistical significance was assessed using the Student’s paired *t*-test. Differences with a *p*-values of <0.05 were considered statistically significant. Datasets of survival curves were analyzed using the Kaplan–Meyer log-rank test. Statistical analyses were conducted using GraphPad Prism 4 software.

## Results

### Rv0652 enhances the expression of surface molecules and induces maturation of functional DCs via TLR4 signaling

**Figure 1:**
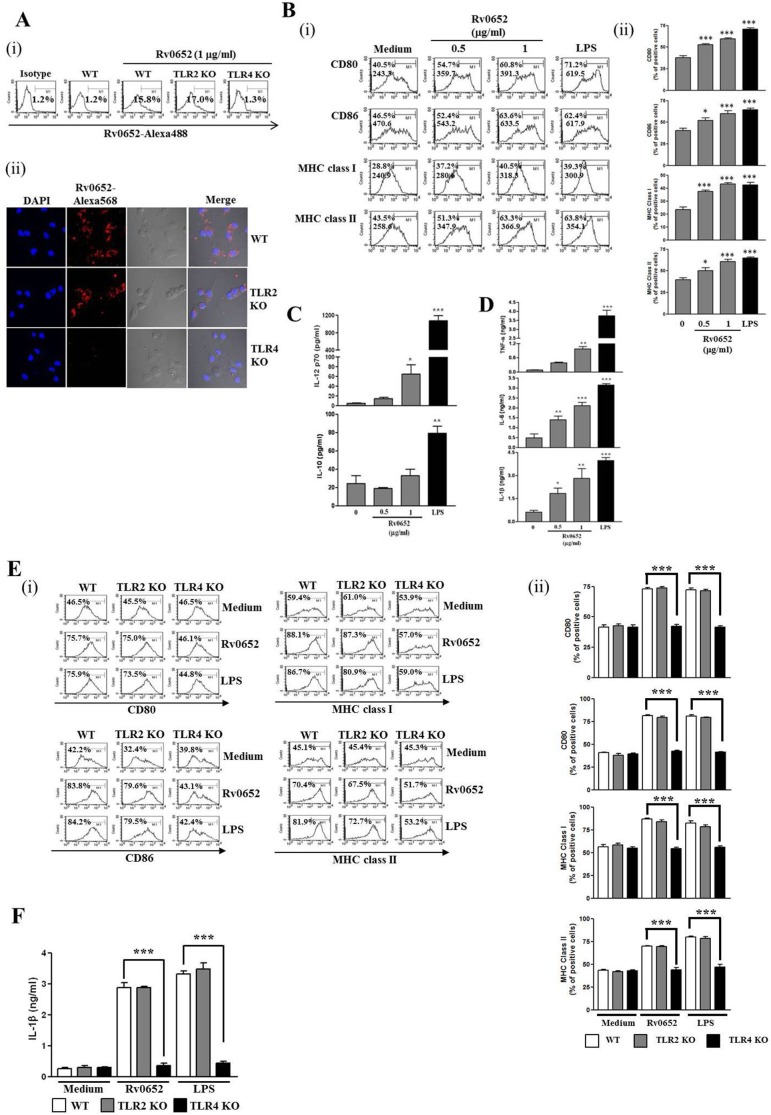
Rv0652 binds to TLR4 but not TLR2, and induces phenotypic and functional maturation of DCs. (A) Fluorescence microscopic observation of Rv0652 binding on DCs. (i) DCs derived from WT, TLR2^−/−^, and TLR4^−/−^ mice were treated with Rv0652 (0.5 ug/ml) for 1 h and stained with Alexa568 h and stained with Alexa568-conjugated anti-Rv0652 mAb and FITC-conjugated CD11c^+^ Ab. The percentage of labeled cells was analyzed by flow cytometry. The percentage of positive cells is shown in each panel. Bar graphs show the mean and standard error of the mean (SEM) for the percentage of Rv0652-Alexa568 labeled CD11c^+^ cells observed in 3 independent experiments. ***P<0.001 compared to Rv0652-treated WT DCs. (ii) DCs derived from WT, TLR2^−/−^, and TLR4^−/−^ mice were treated with Rv0652 (0.5 ug/ml) for 1 h, fixed, and stained with DAPI and Alexa568 h, fixed, and stained with DAPI and Alexa568-conjugated anti-Rv0652 mAb. One representative experiment out of 3 with similar results is shown. (B) Flow cytometry was used to analyze the expression of surface molecules on CD11c^+^ cells. The mean fluorescence intensity (MFI) and the percentage of positive cells are shown in each panel (i). Bar graphs show the percentage of surface molecule-expressing CD11c^+^ cells, representing 5 independent experiments. *P<0.05, **P<0.01, and ***P<0.001 compared to medium control (ii). (C, D) ELISA was performed to test IL-12p70, IL-10, TNF-alpha, IL-6, and IL-1beta production in Rv0652- and LPS-treated DCs. The presented data are the mean and SEM of 3 experiments. *P<0.05, **P<0.01, and ***P<0.001 compared to untreated DCs. (E–F) DCs derived from WT, TLR2^−/−^, and TLR4^−/−^ mice were treated with Rv0652 (1 ug/ml) or LPS (200 ng ng/ml) for 24 h. (E) Flow cytometry was used to analyze the expression of surface molecules on CD11c^+^ cells. The percentage of positive cells is shown in each panel (i). Bar graphs show the percentage of surface molecule-expressing CD11c^+^ cells, representing 4 independent experiments. *P<0.05, **P<0.01, and ***P<0.001 compared with WT cultures (ii). (F) The supernatants were harvested and an ELISA was performed to test IL-1beta production. Data are means and SEM of 3 experiments. ***P<0.001, compared to WT cultures.

Various microbial antigens are recognized by receptors on DCs including TLRs. Signaling by TLRs on DCs after antigen recognition is required to induce an adequate host immune response [14], [15]. To identify which DC TLRs are involved in recognition of Rv0652, we first tested TLR2 and TLR4. DCs from WT, TLR2^−/−^, or TLR4^−/−^ mice were stimulated with Rv0652 (0.5 ug/ml), and Rv0652 was detected on the cell surface with Alexa568-conjugated anti-Rv0652 monoclonal antibodies. Rv0652 bound to the surfaces of WT and TLR2^−/−^ DCs, but not TLR4^−/−^ DCs, (Fig. 1A-(i) and 1A-(ii)).

During maturation, DCs upregulate surface molecules and acquire the ability to produce a broad variety of cytokines, including IL-12 [1]. To determine whether Rv0652 can affect to DC maturation, the surface molecules expressed on DCs after stimulation by Rv0652 were measured. Upregulation of surface molecules was dose-dependent in Rv0652-stimulated DCs (Rv0652-DCs) (Fig. 1B). Cytokine production in Rv0652-DCs was significantly increased, especially IL-12p70, a key cytokine for the polarization of T cells toward the Th1 phenotype, while IL-10, a cytokine with a critical role in Th2 polarization, was produced by Rv0652-DCs at a similar level to that in untreated DCs (Fig. 1C). This suggests that Rv0652 could be used as an adjuvant for the induction of Th1 immune response when using DC vaccines. Rv0652 stimulation also induced high levels of pro-inflammatory cytokine production, such as TNF-alpha, IL-6, and IL-1beta (Fig. 1D) and significantly decreased the capacity of antigen uptake in DCs. The capacity for antigen uptake is decreased during DC maturation that occurs after antigen recognition and uptake, a phenomenon that is similar to what is observed after LPS treatment of DCs (data not shown). These results indicate that Rv0652 significantly enhanced both the phenotypic and functional maturation of DCs.

To analyze the ability of Rv0652 to activate DCs via TLR4, the expression of surface molecules and IL-1beta production were compared in Rv0652-treated WT, TLR2^−/−^, and TLR4^−/−^ DCs. Surface molecule (Fig. 1E) and IL-1beta production (Fig. 1F) were increased in WT and TLR2^−/−^ DCs, but such effects were strongly diminished in TLR4^−/−^ DCs. These results indicated that Rv0652 was a ligand for TLR4 on DCs, and that binding of Rv0652 to TLR4 significantly increased the expression of proteins that are markers for mature DCs and induced functional maturation. The surface markers and signaling pathways induced by TLR4 in DCs are similar to those identified when assessing the role of TLR4 signaling in macrophages [33].

### The effect of Rv0652 purity and endotoxin contamination on cell viability

**Figure 2:**
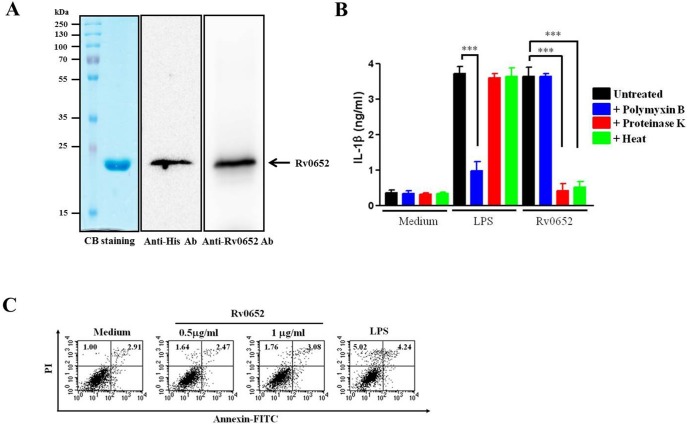
Purity and endotoxin contamination of the rRv0652 protein and its effect on cell viability. (A) Recombinant Rv0652 protein was purified with NTA resin, subjected to SDS-PAGE, and analyzed by using Coomassie blue (CB) staining (left panel) and immunoblot assay using anti-histidine and anti-Rv0652 antibodies (right panel). (B) DCs were stimulated with vehicle, Rv0652 (1 ug/mL), or LPS (200 ng ng/mL), and treated with/without polymyxin B (10 ug/mL), proteinase K (5 ug/ml), or heat (100°C for 30 min min). Culture supernatants were harvested after 24 h, and IL h, and IL-1beta levels were assessed by an ELISA. Data are the mean and SEM of 3 experiments. ***P<0.001. (C) DCs were treated with the indicated concentrations of Rv0652 or LPS (200 ng ng/mL) for 24 h, stained with Annexin V and PI, and analyzed by flow cytometry. Results are representative of 4 separate experiments with similar results. h, stained with Annexin V and PI, and analyzed by flow cytometry. Results are representative of 4 separate experiments with similar results.

Soluble rRv0652 was carefully purified from in *E. coli* under endotoxin-free experimental conditions. Using the LAL endotoxin assay kit (GenScript USA, Inc, Piscataway, NJ, USA), we confirmed that endotoxin contamination of rRv0652 did not occur (<15 pg/ml) (Data not shown). As shown in Fig. 2A, the purity of rRv0652 was analyzed using an immunoblot with anti-histidine and an anti-Rv0652 Abs. The apparent molecular mass of purified rRv0652 was approximately 22 kDa. Recombinant Rv0652 is composed of 130 amino acids, as shown in Supplementary Figure1. Proteinase K- or heat-treated rRv0652 lost the ability to stimulate IL-1beta production in DCs. The activity of rRv0652 was resistant to polymyxin B treatment, indicating that LPS contamination was not responsible for the observed effects (Fig. 2B).

Next, we examined the effect of rRv0652 on the viability of DCs by performing FACS analysis of PI/Annexin V stained cells. As shown in Fig. 2C, rRv0652-DCs at concentrations up to 1 ug/ml had no effect on cell viability, indicating that the purity of rRv0652 was sufficient for use in DC maturation induction. The activation of TLR4 signaling in DCs by rRv0652 recognition, and the induced maturation of DCs, did not affect cell viability when less than 1 ug/ml rRv0652 was used.

### MyD88- and TRIF-signaling pathways contribute to pro-inflammatory cytokine production in Rv0652-DCs

**Figure 3:**
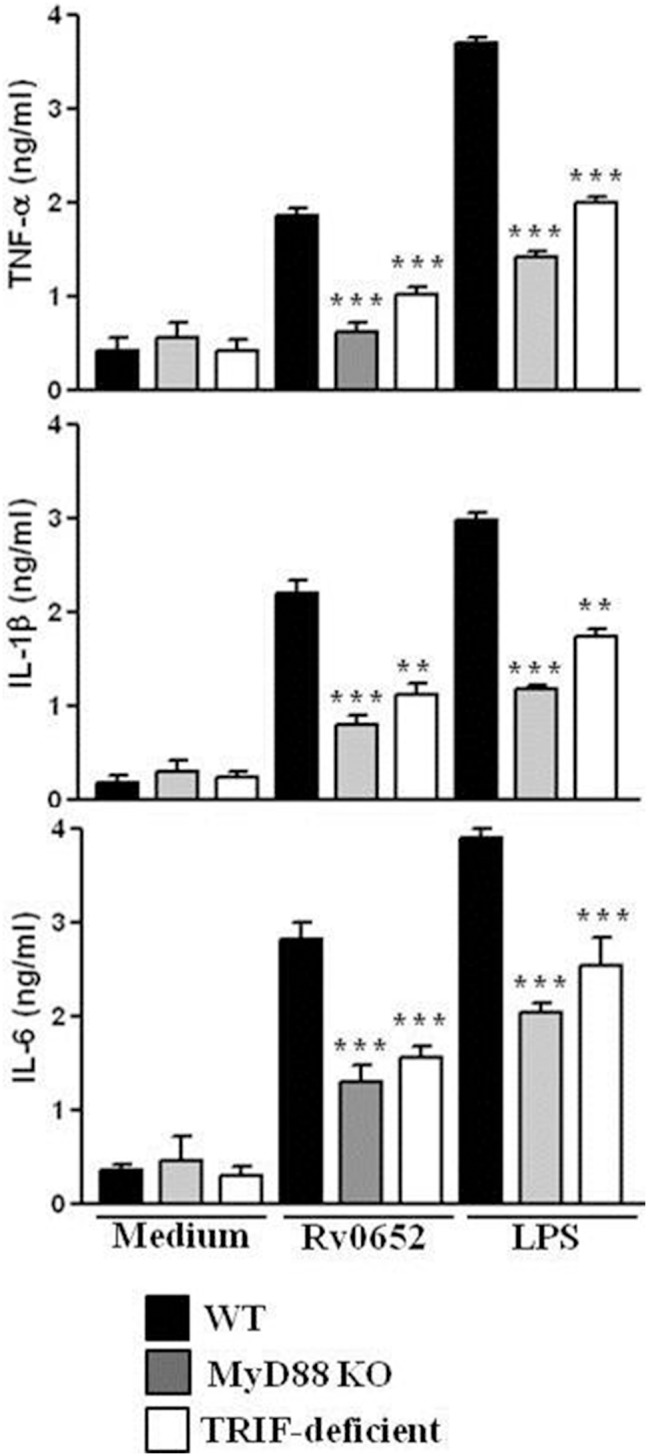
Rv0652-induced cytokine production is partially mediated by MyD88- and TRIF-pathways. DCs derived from WT, MyD88^−/−^, and TRIF-deficient mice were treated with Rv0652 (1 ug/ml) or LPS (200 ng ng/ml) for 24 h. The supernatants were harvested and ELISA was performed to test TNF h. The supernatants were harvested and ELISA was performed to test TNF-alpha, IL-6, and IL-1beta production. Data are means and SEM of 3 experiments. **P<0.01 and ***P<0.001, compared to WT cultures.

The production of pro-inflammatory cytokines, especially IL-1beta, by Rv0652-DCs was induced through the TLR4 signaling pathway (Fig. 1F). TLR4 signaling typically proceeds via MyD88- and TRIF-dependent pathways [34]. To investigate the involvement of the MyD88- and TRIF-signaling pathways on cytokine production in Rv0652-DCs, pro-inflammatory cytokine production in Rv0652-DCs from WT, MyD88^−/−^ and TRIF-deficient mice were compared. The rRv0652-induced production of IL-1beta, IL-6, and TNF-alpha was partially inhibited in the absence of MyD88 and TRIF (Fig. 3). Taken together, our results suggest that TLR4, MyD88, and TRIF are involved in optimal rRv0652-induced pro-inflammatory cytokine responses in DCs.

### rRv0652 induces DC migration

**Figure 4:**
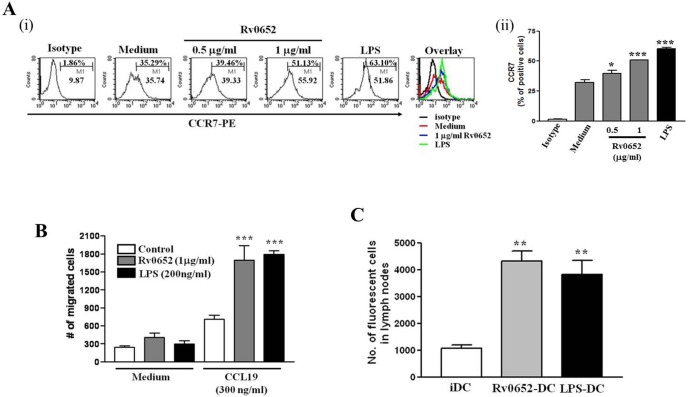
Rv0652 enhances the migration of DCs *in vitro* and *in vivo*. (A) DCs were treated with the indicated concentrations of Rv0652 or LPS (200 ng ng/ml) for 24 h. The percentage of CCR7 h. The percentage of CCR7^+^CD11c^+^ DCs was analyzed by flow cytometry. The number in each panel indicates the percentage of positive cells (i). Bar graphs show the percentage of CCR7-expressing CD11c^+^ cells, representing 4 independent experiments. *P<0.05 and ***P<0.001 compared to the media control (ii). (B) DCs were treated with Rv0652 or LPS for 24 h and then subjected to an *in* *vitro* transwell chemotaxis assay using media alone or media containing CCL19 (300 ng ng/ml). Recorded data are the mean and SEM of 4 experiments. ***P<0.001, compared to untreated DCs. (C) CFSE-labeled Rv0652 (1 ug/ml)- or LPS (200 ng ng/ml)-treated DCs were injected subcutaneously into the hind leg footpad of mice, the cells were recovered from popliteal LNs 72 h later and then analyzed. Data are the mean and SEM of 4 experiments. **P<0.05 compared to untreated DCs.

CCR7, a receptor important for the migration of activated DCs to a secondary lymphoid organ after antigen recognition and uptake in the peripheral tissue, was expressed in DCs during maturation [35],[36]. To investigate whether rRv0652 affects DC migration, CCR7 expression was measured on DCs. CCR7 expression was increased in Rv0652-DCs, to levels similar to those of DCs treated with LPS at 1 ug/ml (Fig. 4A). The migration of DCs in response to CCL19 was examined *in* *vitro* by using a transwell migration assay. Increased migration of DCs was observed (Fig. 4B), consistent with the upregulation of CCR7 expression. To further confirm the migratory capacity of Rv0652-DCs, we investigated the number of CFSE-positive DCs in the draining LNs (dLNs) *in* *vivo*. The number of CFSE-positive DCs in the dLNs was significantly higher in the Rv0652-treated groups compared to those lacking Rv0652 treatment (Fig. 4C). Overall, the migration of DCs from the periphery to secondary lymphoid organs is crucial for their interaction with other immune cells, especially T cells, and the induction of a cellular immune response for cancer immunotherapy. These results demonstrate that rRv0652 promotes DC migration *in* *vitro* and *in* *vivo*, and they suggest antigen-specific T cell responses might be induced by Rv0652-DCs.

### Rv0652-DCs enhance CD8^+^ and CD4^+^ T cell proliferation

**Figure 5:**
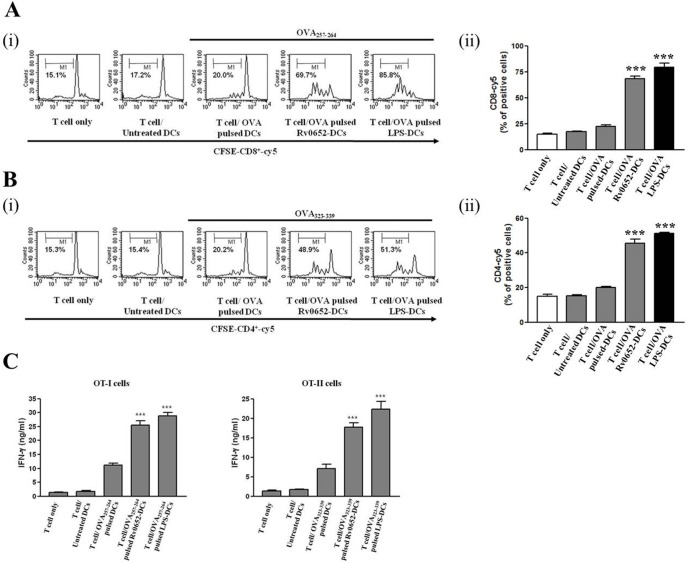
Rv0652-treated DCs induce proliferation of CD4^+^ and CD8^+^ T cells and a Th1 response. Transgenic OVA-specific CD8^+^ T cells (A-i) and OVA-specific CD4^+^ T cells (B-i) were isolated and stained using CFSE. These cells were then co-cultured for 96 h with untreated DCs, DCs pulsed with OVA h with untreated DCs, DCs pulsed with OVA_257–264_, Rv0652 (1 ug/ml)-treated DCs pulsed with OVA_257–264_, or LPS (200 ng ng/ml)-treated DCs pulsed with OVA_257–264_. T-cell proliferation was assessed using flow cytometry and the percentage of proliferating cells is shown in each panel. Bar graphs show the percentage of OVA-specific CD8^+^ T cells (A-ii) and OVA-specific CD4^+^ T cells (B-ii) from 3 independent experiments. ***P<0.001 compared to T cells/OVA pulsed-DCs. (C) IFN-gamma production was measured in each culture supernatant from Fig. 5A and 5B after 24 h of culture by performing an ELISA. Data are the mean and SEM of 3 experiments. ***P<0.001 compared to the value from T cell/OVA peptide-pulsed DCs.

A major role of mature DCs is antigen presentation to T cells, and subsequent T cell activation. To characterize the effect of Rv0652 on DC and T-cell interactions, we performed a syngenic mixed lymphocyte reaction assay by using OT-I T cell receptor (TCR) transgenic CD8^+^ T cells and OT-II TCR transgenic CD4^+^ T cells [37]. Co-culture of CD4^+^ or CD8^+^ T cells (CFSE-labeled and OVA-specific) with Rv0652-DCs pulsed with OVA_257–264_ (Fig. 5A) or OVA_323–339_ (Fig. 5B) induced a significantly higher rate of proliferation than the co-culture of CD4^+^ or CD8^+^ T cells (CFSE-labeled and OVA-specific) with DCs that were pulsed with OVA_257–264_ or OVA_323–339_, but not stimulated with rRv0652. Syngeneic CD4^+^ and CD8^+^ T cells primed with Rv0652-DCs produced significantly (*P*≤0.05–0.01) higher levels of IFN-gamma production than those primed with iDCs (Fig. 5C). These results suggest that rRv0652 induces DC maturation and production of IL-12p70 from DCs. Rv0652-DCs could be used in cancer immunotherapy to indirectly potentiate CD4^+^ T cell differentiation towards a Th1 phenotype, leading to the production of IFN-gamma and direct activation of OVA-specific CD8^+^ T cells.

### Rv0652 enhances the efficacy of DC-based antitumor immunotherapy

**Figure 6:**
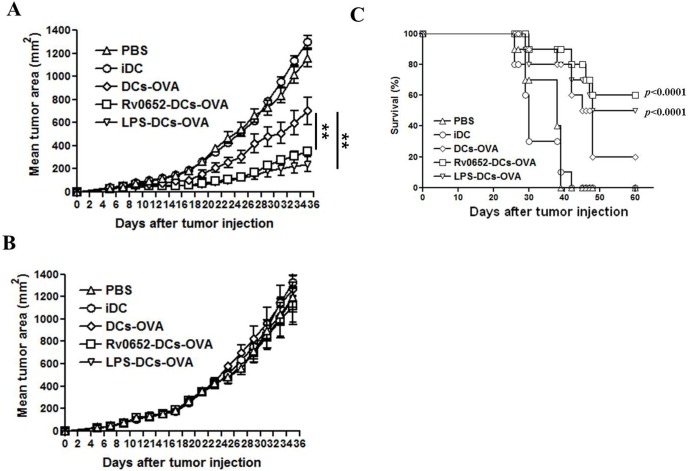
Rv0652-treated DCs pulsed with OVA_257–264_ protects mice against E.G7 tumor challenge. C57BL/6 mice were challenged s.c with 3×10^5^ E.G7 or EL4 tumor cells into the right flank area. For administration of DCs (1×10^6^ cells/mice), mice were injected intravenously with PBS, iDC, DCs-OVA (DCs pulsed with OVA_257–264_), Rv0652-DCs-OVA (Rv0652-treated DCs pulsed with OVA_257–264_), or LPS-DCs-OVA (LPS-treated DCs pulsed with OVA_257–264_) on days 1, 3 and 5 after the tumor challenge. Following the tumor challenge with E.G7 (A) and EL4 (B), tumor growth was monitored by measuring the diameter of the tumor every 2 days. **P<0.01, each group, n = 10. (C) Survival of mice with an E.G7 tumor challenge after injection of OVA peptide-pulsed and Rv0652-treated DCs, n = 10 mice/group. *P* value calculated by Kaplan-Meyer log-rank test between two groups of mice injected with DCs-OVA and Rv0652-DCs-OVA, respectively.

Antigen-specific T cell activation by Rv0652-DCs was observed (Fig. 5); therefore, a mouse tumor model implanted with EL4 or OVA-expressing E.G7 cells was used to determine the potential usefulness of an Rv0652-DC antitumor vaccine. DCs pulsed with OVA_257–264_ (OVA-DCs) and injected intravenously resulted in a significantly slower growth of E.G7 tumors than intravenous injection of PBS or iDCs (Fig. 6A), but did not affect EL4 tumor growth (Fig. 6B). Interestingly, E.G7 tumors in mice that received Rv0652-DCs pulsed with OVA_257–264_ (Rv0652-OVA-DCs) and mice that received LPS-DCs pulsed with OVA_257–264_ (LPS-OVA-DCs) as positive control were significantly smaller than E.G7 tumors in mice that received OVA-DCs (Fig. 6A). Notably, only 20% of mice injected with OVA-DCs survived beyond 60 days following E.G7 tumor implantation, while over 60% of mice injected with Rv0652-OVA-DCs survived longer than 60 days. All mice in the PBS and iDC-injected groups had a median survival of 34 days with no long-term survivors (Fig. 6C).

### Rv0652 increases CTL activity in tumor-bearing mice

**Figure 7:**
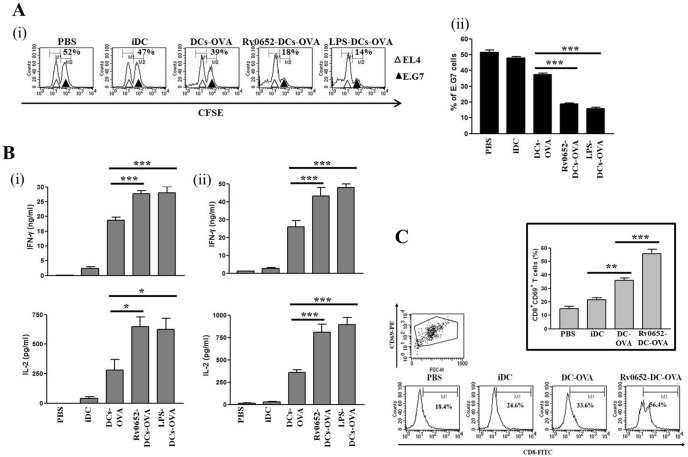
Rv0652-treated DCs pulsed with OVA_257–264_ enhances CTL activity and CD8^+^ T-cell tumor infiltration. C57BL/6 mice were immunized with PBS, iDC, DCs-OVA (DCs pulsed with OVA_257–264_), Rv0652-DCs-OVA (Rv0652-treated DCs pulsed with OVA_257–264_), or LPS-DCs-OVA (LPS-treated DCs pulsed with OVA_257–264_) on days 1 and 7. (A) Splenocytes from the mice immunized with PBS or various DCs for 10 days were treated with OVA_257–264_ (10 ug/ml) for 7 days and then co-cultured with EL4 (CFSE^low^) or E.G7 (CFSE^high^) cells. After 2 days, mixed lymphocyte tumor cultures were analyzed by flow cytometry. Histograms show antigen-specific lysis of tumor cells. The percentage in each panel indicates specific lysis of E.G7 cells (i). Bar graphs show the percentage of specific lysis of E.G7 cells representing 4 independent experiments (ii). ***P<0.001. (B) Splenocytes (i) and tumor infiltrating CD8^+^ T cells (ii) from the mice immunized with PBS or various DCs for 10 days were treated with OVA_257–264_ (10 ug/ml) for 24 h IL h IL-2 and IFN-gamma production was measured in supernatants by ELISA. Data are the mean and SEM of 4 experiments. *P<0.05 and ***P<0.001. (C) C57BL/6 mice were challenged s.c. with E.G7 tumor cells to the right flank area. One, 3, and, 5 days after the tumor challenge, mice were injected intravenously with PBS, iDC, DCs-OVA (DCs pulsed with OVA_257–264_), or Rv0652-DCs-OVA (Rv0652-treated DCs pulsed with OVA_257–264_) (n = 3). At day 20, the cells in the tumor area were analyzed for the presence of CD8^+^CD69^+^ T cells. The percentage in each panel indicates the infiltration of CD8^+^CD69^+^ T cells into the tumor area. Data are the mean and SEM of 3 experiments. **P<0.05 and ***P<0.001.

Rv0652-DCs had an antitumor effect through T cell activation (Fig. 5), inhibition of tumor growth, and extended survival (Fig. 6). To determine whether T cells activated with Rv0652-DCs actually exerted tumor-killing effects, we examined *ex* *vivo* CTL activity and cytokine production, including that of IFN-gamma and IL-2. CTLs from mice injected with OVA-DCs lysed E.G7-OVA target cells more efficiently than CTLs from mice injected with PBS or iDCs (Fig. 7A). CTLs from mice injected with Rv0652-OVA-DCs in E.G7 as well as LPS-OVA-DCs (the positive control) were able to lyse target cells at a significantly greater rate than CTLs from mice injected with OVA-DCs (Fig. 7A). IFN-gamma and IL-2 secretion from splenocytes (Fig. 7B-a) and tumor-infiltrating CD8^+^ T cells (Fig. 7B-b) from mice injected with Rv0652-OVA-DCs and then re-stimulated with OVA_257–264,_ was higher than the IFN-gamma and IL-2 secretion from splenocytes and tumor-infiltrating CD8^+^ T cells from mice injected with OVA-DCs. This indicates that the antitumor effect of Rv0652-OVA-DCs is closely related to the production of IFN-gamma and IL-2, as well as CTL activity (Fig. 7B). These data suggest that rRv0652 acts as a Th1-polarizing adjuvant. We examined whether administration of Rv0652-OVA-DCs enhanced the number of CD8^+^CD69^+^ T cells, the phenotype of activated CD8^+^ T cells. The number of activated CD8^+^ T cells from mice injected with OVA-DCs was higher than that of mice injected with either PBS or iDCs. More importantly, the number of activated CD8^+^ T cells from mice injected with Rv0652-OVA-DCs was greater than that of the mice injected with OVA-DCs (Fig. 7C). These results indicate that Rv0652-OVA-DCs can induce an antitumor immune response by increasing the antitumor activity of CTLs.

## Discussion

The immune system can recognize many tumor Ags; however, the ability of tumors to escape a host immune system suggests that the immune mechanism is insufficient for effectively killing certain tumors. In an attempt to strengthen antitumor immunity, we focused on a DC-based therapeutic strategy, using *M. tuberculosis* Rv0652.

DCs are the most potent APCs and versatile regulators of T lymphocyte responses. Immature DCs capture Ags, process them, and present them on the cell surface, in association with MHC class I or II molecules, to prime CD8^+^ CTLs or CD4^+^ T helper 1 cells, respectively [1]. Immunization with DCs loaded *ex* *vivo* with tumor Ag, is a promising strategy for inducing efficient antitumor immunity [4] that has been successfully used to vaccinate mice and to activate CTL responses in preclinical trials [5]–[8].

We showed that rRv0652 could induce antigen specific antitumor effects by triggering DC maturation. Rv0652 improved the efficacy of DC-based antitumor immunotherapy in mice by acting as a potent adjuvant. Evidence for these novel characteristics of rRv0652 include: 1) increased expression of surface molecules (CD80/CD86); 2) involvement of the MyD88- and TRIF-pathways in the production of pro-inflammatory cytokines induced by Rv0652 in DCs; 3) DC activation was not due to endotoxin contamination and occurred via TLR4 but not TLR2 signaling; 4) there is an increased migratory capacity in Rv0652-DCs; 5) the effect of Rv0652-DCs on the expansion of IFN-gamma-producing CD4^+^ and CD8^+^ T cells; and 6) significantly elevated Ag-specific CTL antitumor activity against an E.G7-OVA expressing thymoma.

Co-stimulatory signals, such as CD40, CD80, and CD86 as well as cytokines including IL12p70 from Ag-specific and activated DCs are required for the induction of proper host immunity mediated by helper CD4^+^ and cytotoxic CD8^+^ T cells. We found that Rv0652-DCs significantly increased their surface molecules and the production of IL-12p70, which is essential for Th1 polarization [38]. In addition, the interaction of T cells with Rv0652-DCs led to a higher rate of primary syngenic T cell proliferation than untreated DCs. The activation of T cells is a crucial event for an effective immune response against a tumor, and Rv0652-DCs induced IFN-gamma production in CD4^+^ and CD8^+^ T-cells. The production of IFN-gamma is an important feature of Th1 immune responses, and this result suggests that Rv0652 may promote a Th1 immune response through the activation and maturation of DCs. Furthermore, Rv0652 protein enhanced CCR7 expression, which is crucial for DC migration from the periphery into the paracortex of secondary lymphoid organs [35], [36]. Rv0652-DCs migrate towards CCL19 *in* *vitro* and to draining LNs *in* *vivo*. Therefore, Rv0652 affects DC function in multiple ways, and Rv0652 may be an effective adjuvant for DC-based antitumor immunotherapy.

TLRs are expressed by numerous immune cells and play key roles in innate and adaptive immune responses [39]. Among various TLRs, TLR4 agonism potentially results in more efficient DC maturation and enhances Ag presentation to T cells [40]. Adjuvant studies for cancer vaccine treatments have recently focused on TLR4 agonists because these receptors mediate signals from the innate immune system via MyD88- and TRIF-dependent pathways, and these appear to be important immunopotentiators [41]. Although LPS, the most studied and well-characterized TLR4 agonist, is highly effective as an adjuvant in both experimental and preclinical settings [42], it is not suitable for clinical use due to its toxicity in humans. Thus, novel TLR4 agonists are needed for use as adjuvants in tumor and infectious disease vaccine production, especially when using DC-based vaccination strategies.

Several purified *M. tuberculosis* ligands, such as LprA [25], and PE-PGRS [26] have now been identified as TLR2 agonists that induce APC activation [43]. Our group recently showed that *M. tuberculosis* HBHA is a novel TLR4 agonist for antitumor vaccinations [27]. We also made the novel observation that *M. tuberculosis* Rv0652, similar to HBHA, directly binds TLR4 and activates TLR4 signaling, leading to the expression of co-stimulatory and MHC antigen presentation molecules, as well as the production of IL-1beta by DCs. Kim *et al* [33] have observed similar results in macrophages stimulated with Rv0652. Most information regarding the role of MyD88 and TRIF in mediating cytokine production by TLR4 has come from studies employing LPS and DnaK as TLR4 agonists. Our results suggested that intracellular adapters such as MyD88 and TRIF are partially involved in mediating pro-inflammatory cytokine production in Rv0652-DCs. Although MyD88- and TRIF-mediated signaling is essential for an optimal Rv0652-induced cytokine response by DCs, there is a possibility that other signaling pathways are involved in pro-inflammatory cytokine production, since MyD88^−/−^ and TRIF^−/−^ DCs still produce small amounts of pro-inflammatory cytokines. Further study is necessary to fully understand this process.

Systemic intravenous (Fig. 6) and subcutaneous (Supplementary Fig. 2) injection of Rv0652-treated, OVA peptide-pulsed DCs induced a marked suppression of tumor growth. Using the Systemic intravenous injection model, we observed that the antitumor activity of Rv0652 originates from a marked increase in IFN-gamma production by the T cells in draining LNs that are primed by DCs stimulated with Rv0652, an increase in the number of functional Ag-specific CTLs, and increased tumor infiltration of CD8^+^CD69^+^ T cells. Taken together, this increase in antitumor efficacy was attributed to the enhancement of Th1 polarization by Rv0652-DCsthat activate the CTLs that are able to lyse tumor cells. Our data indicate that *M. tuberculosis* Rv0652 can be used in DC-based antitumor immunotherapy.

## Supporting Information

Figure S1 **Amino acid sequence of Rv0652 (** www.tbdb.org **).**
(TIF)

Figure S2 **Rv0652-treated DCs pulsed with OVA_257–264_ protects mice against E.G7 tumor challenge.** C57BL/6 mice were challenged s.c with 3×10^5^ E.G7 tumor cells into the right flank area. For administration of DCs (1×10^6^ cells/mice), mice were injected subcutaneously with PBS, iDC, DCs pulsed with OVA_257–264_, or Rv0652-treated DCs pulsed with OVA_257–264_ on days 1, 3 and 5 after the tumor challenge. (A) Following tumor challenge with E.G7 tumor growth was monitored by measuring the diameter every 2 days. *P<0.05, each group, n = 10. (B) Survival of mice with E.G7 tumor challenge after injection of OVA peptide-pulsed and Rv0652-treated DCs, n = 10 mice/group. *P* value calculated by Kaplan-Meyer log-rank test between two groups of mice injected with iDC and Rv0652-DC-OVA, respectively.
(TIF)
