# Supplementary figures and images for: A Potential Protein Adjuvant Derived from Mycobacterium tuberculosis Rv0652 Enhances Dendritic Cells-Based Tumor Immunotherapy
Source: PLoS One. 2014 Aug 7;9(8):e104351. doi: 10.1371/journal.pone.0104351 (PMC4125215; doi:10.1371/journal.pone.0104351)

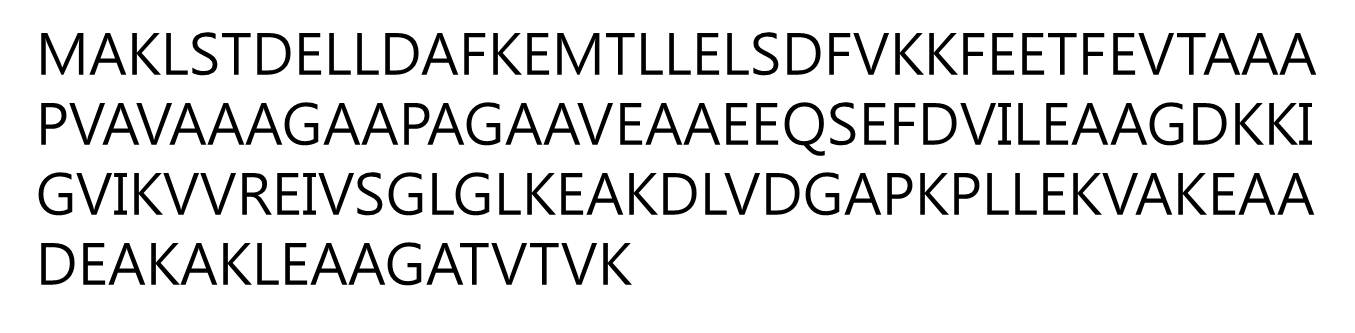

Supplement: Figure S1 — Amino acid sequence of Rv0652 ( www.tbdb.org ). (TIF) [file pone.0104351.s001.tif]

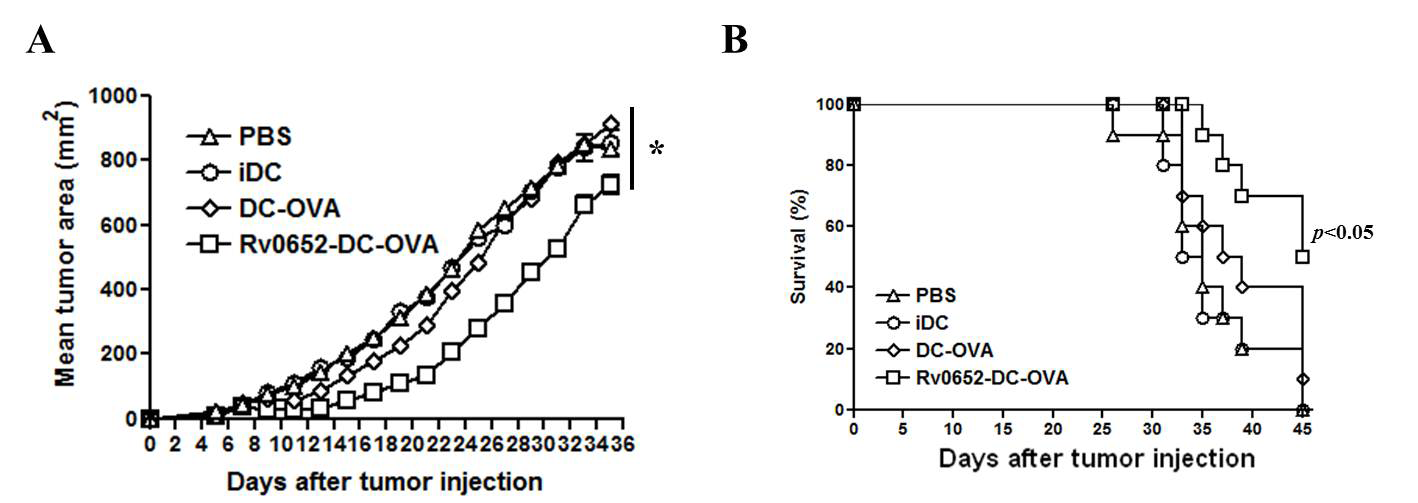

Supplement: Figure S2 — Rv0652-treated DCs pulsed with OVA257–264 protects mice against E.G7 tumor challenge. C57BL/6 mice were challenged s.c with 3×10^5^ E.G7 tumor cells into the right flank area. For administration of DCs (1×10^6^ cells/mice), mice were injected subcutaneously with PBS, iDC, DCs pulsed with OVA257–264, or Rv0652-treated DCs pulsed with OVA257–264 on days 1, 3 and 5 after the tumor challenge. (A) Following tumor challenge with E.G7 tumor growth was monitored by measuring the diameter every 2 days. *P<0.05, each group, n = 10. (B) Survival of mice with E.G7 tumor challenge after injection of OVA peptide-pulsed and Rv0652-treated DCs, n = 10 mice/group. P value calculated by Kaplan-Meyer log-rank test between two groups of mice injected with iDC and Rv0652-DC-OVA, respectively. (TIF) [file pone.0104351.s002.tif]
